# Four new mitochondrial genomes of the genus *zosterops* (aves: passeriformes: zosteropidae) from East Africa with a phylogenetic evaluation of the group

**DOI:** 10.1080/23802359.2016.1198937

**Published:** 2016-08-21

**Authors:** Martin Husemann, Sarah Sturm, Manuel Curto, Harald Meimberg, Jan Christian Habel

**Affiliations:** a Department of General Zoology, Institute of Biology, Martin-Luther University Halle-Wittenberg, Halle, Saale, Germany;; b Department of Ecology and Ecosystem Management, Terrestrial Ecology Research Group, School of Life Sciences Weihenstephan, Technische Universität München, Freising, Germany;; c Centrum Für Naturkunde, Universität Hamburg, Hamburg, Germany;; d Research Center in Biodiversity and Genetic Resources (CIBIO)/InBio Associated Laboratory, University of Porto, Vairão, Portugal;; e Institute for Integrative Nature Conservation Research, University of Natural Resources and Life Sciences (BoKu), Vienna, Austria

**Keywords:** Illumina MiSeq, primer walking, Zosterops abyssinicus, Zosterops poliogaster, Zosterops senegalensis

## Abstract

The white-eye birds of the genus *Zosterops* have been recognized for their high speciation rates in the past, but the relationships of the East African populations are not yet fully resolved. We sequenced and annotated mitogenomes of four populations currently assigned to three East African white-eye species, *Zosterops senegalensis*, *Z. abyssinicus* and *Z. poliogaster*. For *Z. senegalensis* specimens from two distant populations were sequenced; for the other taxa we used samples collected at one site. The mitogenomes ranged between 17,827 and 17,974 bp, in size similar to previously published mitogenomes analyzed for this genus from other geographic regions. The mitogenomes contain the classical set of 13 coding genes, two structural rRNA genes and 22 tRNA genes. We constructed a phylogeny using all complete mitogenomes currently available for the genus. The phylogeny supports an Asian or Oceanic origin of the genus *Zosterops*. The East African species represent a monophyletic clade, but the two specimens of *Zosterops senegalensis* from different regions do not group together, supporting previous hypotheses of cryptic species within the genus. The new genetic resources provided here may help to further explore the relationships and evolution of the genus.

## Introduction

Mitogenomes provide important information for phylogenetic and phylogeographic studies. They are characterized by functional genes with a variety of different evolutionary rates and hence can be used for analyses of taxa with different divergent times (Curole & Kocher 1999; Jacobsen et al. 2012). They have been shown to be highly informative to resolve the basic taxonomic relationships within the Passerine birds (Barker 2014).

The Passerine bird genus *Zosterops* is an interesting target for evolutionary biology as it is characterized by high rates of speciation and a large number of endemic species, especially island endemics (Gill 1971; Moyle et al. 2009; Milá et al. 2010; Cornetti et al. 2015). In East Africa, only three species have been recognized as valid species for a long time (Moreau 1957), but more recently it has become evident that local populations are strongly differentiated from each other (genetically, phenotypically, specifically in regard of plumage coloration and bioacoustic traits) and hence should be treated as distinct taxa (Habel et al. 2013, 2014, 2015; Cox et al. 2014; Husemann et al. 2014, 2015). However, genetic resources for the group are still limited reducing phylogenetic resolution in previous studies.

More recently, several mitogenomes of Zosterops species have become available (*Zosterops lateralis* – Gibb et al. 2015; *Zosterops erythropleurus* – Li et al. 2016; *Zosterops japonicus* – Yang et al. in press). However, so far no (mito)genome of an East African species was sequenced. Hence, we used a hybrid approach of Sanger Sequencing and Illumina MiSeq to generate whole mitochondrial genomes of four populations identified as three species of East African *Zosterops* species. We annotated the genomes and provide a phylogeny based on these and all other published mitogenome sequences of *Zosterops*.

## Material and methods

We included 17 individuals of *Z. senegalensis* collected at two sites, Kakamega Forest (0°17’ N; 34°50’ E) (1 individual for Sanger sequencing and 7 individuals for Illumina MiSeq), and Mt. Nyeri (2°05’ N, 36°42’ E) (1 individual for Sanger sequencing and 8 individuals for Illumina MiSeq); 5 individuals of *Z. poliogaster* were collected in the Chyulu Hills (2°36’ S; 37°50’ E) (2 individuals for Sanger sequencing, 3 individuals for Illumina MiSeq); 12 individuals of *Z. abyssinicus* were collected at the foothills of the Chyulu Hills (2°25’ S; 37°55’ E) (with 2 individuals for Sanger sequencing and 10 individuals for Illumina MiSeq). Birds were caught with mist nets and a blood sample was taken and stored in 99% ethanol. The birds were subsequently released unharmed.

DNA was extracted from blood using the Qiagen DNeasy blood and tissue kit (Qiagen, Hilden, Germany) using the manufacturers protocol for blood. DNA extracts are stored at the Technical University Munich under the following internal accession numbers: *Z. senegalensis* – Kakamega Forest: T41, T42, T49, T50-T54; Mt. Nyeri: T58, T71-T76, T82, T83; *Z. poliogaster*: CH1, CH2-1, CH2-2, CH2-3, CH2-4; *Z. abyssinicus*: T05-T12, T17-20. PCRs were performed with standard protocols.

**Figure 1.**
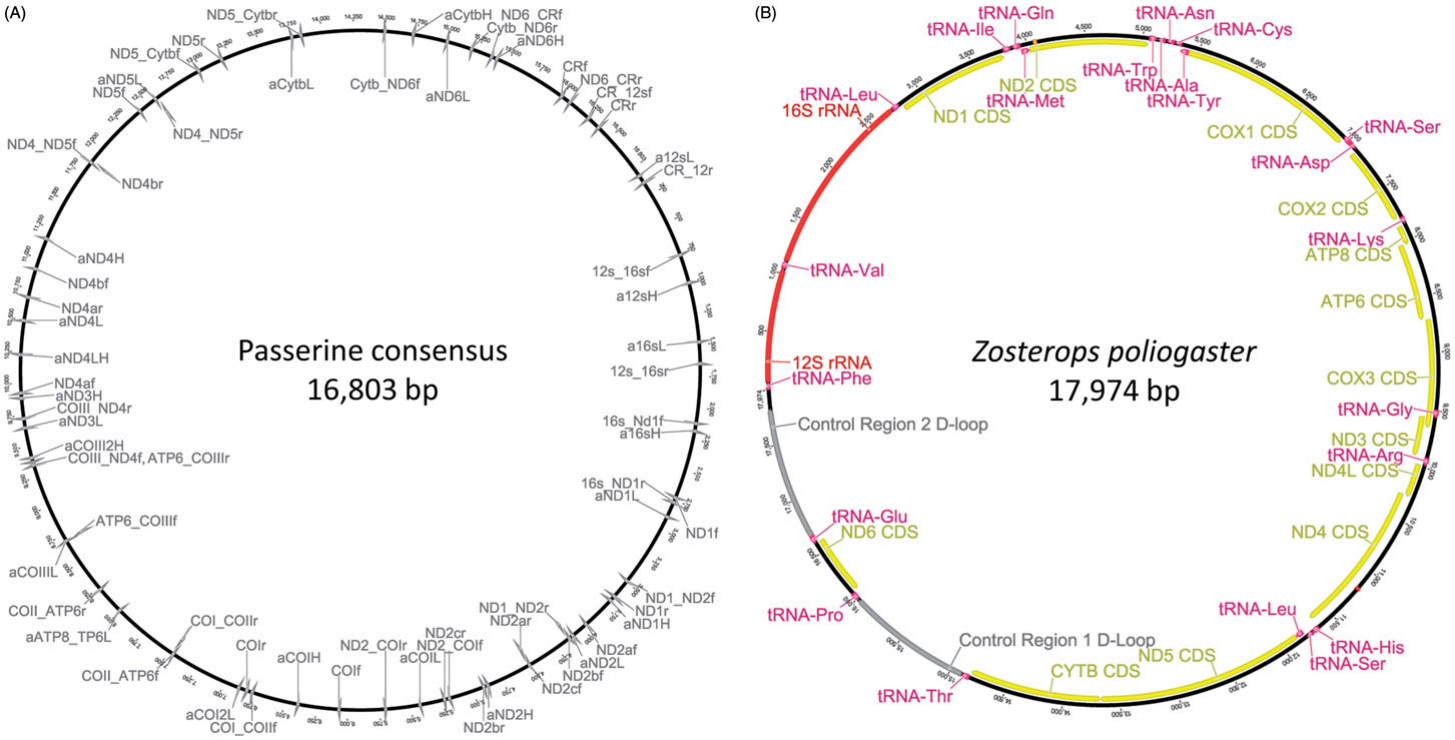
(A) Primer binding sites of all primers for Sanger sequencing used in this study (primer sequences available from the authors upon request) mapped against the consensus sequence of 41 published Passerine mitogenomes. (B) Annotated mitogenome of *Zosterops poliogaster* displaying gene order and composition; red - rRNA genes, pink - tRNA genes, yellow - coding genes, grey - Control Region.

We initially sequenced 13 mitochondrial gene fragments using the primers provided by Amer et al. (2013). Their primer set lacked primers for the Control Region and the primers for ND5, COII and COIII did not yield good results. The lack of primer binding was further confirmed by mapping all primers against a consensus sequence derived from 41 Passerine mitogenomes obtained from Genbank (Figure 1A, Accessions: KF509924, NC020429, JQ348398, NC015114, HQ690245, NC020427, JQ423933, NC015233, HQ896034, NC015196, HQ915865, NC015198, HQ915866, NC015232, HQ896033, NC015802, JN018411, NC015074, HQ690246, NC015898, JF937590, NC020423, JQ003191, JQ003192, NC015237, HQ896037, NC015613, JF810423, NC015195, HQ915864, NC020424, JQ083495, NC020426, JQ423932, NC015824, JN108020, KF509923, NC015810, JN018413, NC015200, HQ915867). Therefore, we designed a new set of 22 primer pairs based on the consensus sequence and the preliminary assemblies for *Zosterops* derived from the initial primer set (available from the authors upon request, Figure 1A).

The newly generated sequences were trimmed and *de-novo* assembled using Geneious v. 6.1.8 (Kearse et al. 2012). For this, sequences from individuals belonging to the same populations were pooled. The resulting contigs were mapped against the published sequences of *Z. lateralis* (KC545407) to generate an initial assembly. The assemblies were further refined using Illumina MiSeq reads of specimens from identical populations (see above), which were generated for a different study with a new amplicon-based protocol that is described elsewhere (Meimberg et al. 2016). The final alignments were initially annotated using MitoAnnotator (Iwasaki et al. 2013) and Mitos (Bernt et al. 2013). The initial annotation was further refined by comparison to other published mitogenomes of *Zosterops* species (*Z. lateralis* – KC545407, *Z. erythropleurus* – KT194322, *Z. japonicus* – KT601061). Finally, we adjusted annotations of coding genes to implement stop codons, which were not implemented in the published mitogenomes for two of the genes.

We used the new mitogenomes together with published sequences to generate a phylogenetic tree with MrBayes v.3.2.6 (Ronquist et al. 2012). We included the newly generated sequences for *Z. abyssinicus* from the Chyulu Hills foothills, *Z. poliogaster* from the Chyulu Hills, and sequences for *Z. senegalensis* from Kakamega Forest and Mt. Nyeri. We further included all other available sequences for *Zosterops* species (*Z. lateralis* – KC545407, *Z. erythropleurus* – KT194322, *Z. japonicus* – KT601061) and used the consensus sequence of Passerines (see above) as outgroup. The best model of sequence evolution was determined as GTR + G by MrModeltest v.2 (Nylander 2004). We ran Mr Bayes for 10 million generations sampling every 1000 generations for a total of 10,000 sampled trees. Convergence was checked by the average split frequencies which were below 0.001. The tree was visualized using FigTree v.1.4.2 (http://tree.bio.ed.ac.uk/software/figtree/).

## Results and discussion

We generated mitogenomic sequences for four populations of East African *Zosterops*: two populations of *Z. senegalensis* from Kakamega Forest and Mt. Nyeri, *Z. poliogaster* from the Chyulu Hills and *Z. abyssinicus* from the foothills of the Chyulu Hills. The four newly generated mitogenomes varied in size between 17,827 and 17,974 bp, similar to other mitogenomes published for this genus. They contain the classical set of 13 coding genes, two structural rRNA genes and 22 tRNA genes (Figure 1B). The tRNAs for Leucine and Serine were present in two copies. Similar to other *Zosterops* species we detected two Control Regions. The mitogenomic gene order was similar across all *Zosterops* which have been analyzed so far. The GC content varied between 44.5% for *Z. senegalensis* from Kakamega forest and 45.6% for *Z. poliogaster*. All newly generated sequences were deposited in NCBI GenBank (Accession Numbers: KX181885- KX181888).

**Figure 2.**
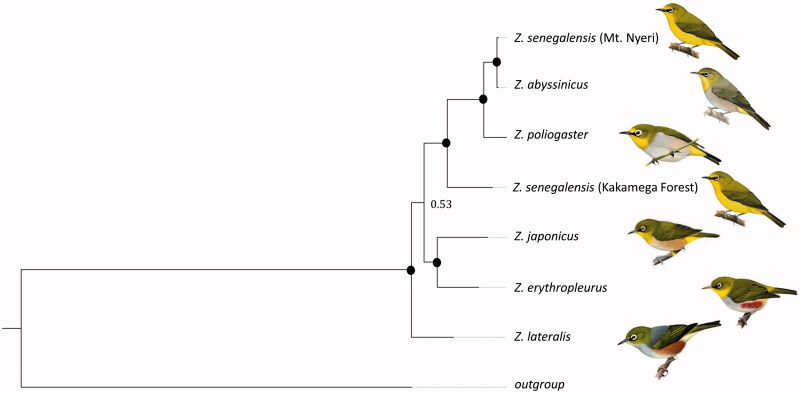
Bayesian phylogenetic tree generated from all available *Zosterops* mitogenomes; black circles indicate posterior probabilities of 1.

Most relationships were strongly supported in our phylogenetic reconstruction (Figure 2; posterior probabilities >0.95). *Zosterops lateralis* was the basal species in the phylogeny. *Zosterops japonicus* and *Z. erythropleurus* were sister taxa and represented the sister group to the East African species. This was the only relationship with no support (pp =0.53). This may be due to limited sampling or saturation effects. The Eastern African species were monophlyletic with maximum support. However, *Zosterops senegalensis* was polyphyletic with the population from Kakamega Forest being basal to all other species and the population from Mt. Nyeri being sister to *Z. abyssinicus*. *Zosterops poliogaster* was the sister taxon to this grouping.

The phylogeny provides further support to previous findings in regard of the Asian or Oceanic origin of the genus (Moyle et al. 2009; Cox et al. 2014). Further, our findings support the non-monophyly of the currently established East African species of the genus, which had been suggested on the basis of morphological, acoustic, microsatellite and sequence data (Habel et al. 2013, 2014, 2015; Cox et al. 2014; Husemann et al. 2014, 2015).
